# Cost-effectiveness of a hypertension management programme in an elderly population: a Markov model

**DOI:** 10.1186/1478-7547-9-4

**Published:** 2011-04-05

**Authors:** Gastón Perman, Emiliano Rossi, Gabriel D Waisman, Cristina Agüero, Claudio D González, Carlos L Pallordet, Silvana Figar, Fernán Gonz&#225lez Bernaldo de Quirós, JoAnn Canning, Enrique R Soriano

**Affiliations:** 1 Medical Programmes, Hospital Italiano de Buenos Aires, (Perón 4253, 2°), Ciudad de Buenos Aires, (C1199ABC), Argentina; 2 Epidemiology Section, Internal Medicine Department, Hospital Italiano de Buenos Aires, (Perón 4253, 2°), Ciudad de Buenos Aires, (C1199ABC), Argentina; 3 Hypertension Section, Internal Medicine Department, Hospital Italiano de Buenos Aires, (Perón 4190, 2°), Ciudad de Buenos Aires, (C1199ABB), Argentina; 4 Financial Department, Hospital Italiano de Buenos Aires, (Perón 4253, 2°), Ciudad de Buenos Aires, (C1199ABC), Argentina; 5 Pharmacology Department, School of Medicine, Universidad Austral, (Perón 1500), Derqui, (B1629AHJ), Provincia de Buenos Aires, Argentina; 6 Fundación Capital, (Sinclair 3088), Ciudad de Buenos Aires, (C1425FRD), Argentina; 7 Strategic Management, Hospital Italiano de Buenos Aires, (Perón 4190, PB), Ciudad de Buenos Aires, (C1199ABB), Argentina; 8 Health Informatics Department, Hospital Italiano de Buenos Aires, (Perón 4272, 3°), Ciudad de Buenos Aires, (C1199ABD), Argentina

## Abstract

**Background:**

Mounting evidence shows that multi-intervention programmes for hypertension treatment are more effective than an isolated pharmacological strategy. Full economic evaluations of hypertension management programmes are scarce and contain methodological limitations. The aim of the study was to evaluate if a hypertension management programme for elderly patients is cost-effective compared to usual care from the perspective of a third-party payer.

**Methods:**

We built a cost-effectiveness model using published evidence of effectiveness of a comprehensive hypertension programme vs. usual care for patients 65 years or older at a community hospital in Buenos Aires, Argentina. We explored incremental cost-effectiveness between groups. The model used a life-time framework adopting a third-party payer's perspective. Incremental cost-effectiveness ratio (ICER) was calculated in International Dollars per life-year gained. We performed a probabilistic sensitivity analysis (PSA) to explore variable uncertainty.

**Results:**

The ICER for the base-case of the "Hypertension Programme" versus the "Usual care" approach was 1,124 International Dollars per life-year gained. PSA did not significantly influence results. The programme had a probability of 43% of being dominant (more effective and less costly) and, overall, 95% chance of being cost-effective.

**Discussion:**

Results showed that "Hypertension Programme" had high probabilities of being cost-effective under a wide range of scenarios. This is the first sound cost-effectiveness study to assess a comprehensive hypertension programme versus usual care. This study measures hard outcomes and explores robustness through a probabilistic sensitivity analysis.

**Conclusions:**

The comprehensive hypertension programme had high probabilities of being cost-effective versus usual care. This study supports the idea that similar programmes could be the preferred strategy in countries and within health care systems where hypertension treatment for elderly patients is a standard practice.

## Background

Over the last three decades, clinical research has shown that effective hypertension treatment lowers cardiovascular events and related deaths[1-12]. In spite of this medical benefit there is increasing worldwide concern about the economic burden of hypertension and associated cardiovascular outcomes[13].

Mounting evidence shows that multi-intervention programmes are more effective than an isolated pharmacological strategy[14-19]. Special attention is being given to "full-service disease management programs",[20] with its key characteristics based on: population identification processes; evidence-based practice guidelines; collaborative practice models; patient self-management education; process and outcome measurement, evaluation and management; and routine reporting/feedback.

Full economic evaluations of hypertension management programmes are scarce[21-24] and contain methodological limitations. These limitations include: short-term analysis; lack of hard outcome measures; exclusive use of secondary databases; and/or deficiencies in sensitivity analysis.

Most economic evaluations in hypertension have focused on the comparison of two drug treatments. The major problem with these evaluations is that they offer little direction to decision makers related to what kind of health services to provide. They address questions limited to a few treatment options for only one aspect -pharmacologic- of hypertension treatment. Moreover, analysis has been primarily based on clinical trials that analyze efficacy in ideal settings not real-life effectiveness.

In year 2000, we started a multidisciplinary antihypertensive programme for elderly patients at Hospital Italiano de Buenos Aires in Argentina. Its effectiveness was demonstrated elsewhere[14]. In this study we evaluate if our hypertension management programme is cost-effective compared to usual care from the perspective of a third-party payer.

## Methods

### Description of different treatment options

The effectiveness of a hypertension management program in middle-class patients 65 years or older was determined by a quasi-experimental, individual-based study[14] with a control group. This study had been previously approved by an Ethics Committee. We compared the intervention -"Hypertension Programme"- against "Usual care" -the control group- using a pragmatic design (i.e. the study was designed to capture the effects of interventions as they were usually performed, avoiding artificial changes due to research protocol).

"Usual care" consisted of attention by primary care physicians (PCP). Visits to the PCP could be on a regular basis or whenever the patient asked for an appointment. There were no restrictions regarding studies, pharmacological treatments or specialty consultations -cardiologists, neurologists, etc., if the PCP agreed with them.

The new "Hypertension Programme" consisted of usual care described above plus: personal and telephone contact with patients by medical students; support with non-pharmacological treatment such as diet and physical activity; educational material and optional workshops focused on patient empowerment and self-efficacy; information recorded on an electronic health record that served as a link among health care workers.

Differences in systolic blood pressure (SBP) level and in percentage of well-controlled (< 140/90 mm Hg) patients between groups were measured at baseline and after 12 months of follow-up. Data were assessed by intention-to-treat analysis. Two hundred and fifty patients were evaluated in each group. There were no baseline differences between intervention and usual care groups besides age (73 vs. 72 years, respectively; p < 0.001; see Additional file 1, appendix). At baseline, mean blood pressure (systolic/diastolic) in mm Hg (SD) was 138(20)/75(11) vs. 135(19)/75(11); and percentage of well-controlled patients was 56.4% vs. 60.4%, respectively. At the end of the study period, the difference of mean change in systolic blood pressure between groups was 7.1 mm Hg (95% confidence interval, 4-10 mm Hg). Sixty-seven percent of patients in the intervention group were well-controlled, versus 51% of patients in the control group (p < 0.001). With these improved results the program was implemented in the whole population of hypertensive patients in the HMO. We used this information to build our model.

### Model construction

Even though we had patient-level data to perform a cost-effectiveness analysis, we decided to build a theoretical model that considered these data because we could not track long-term costs and/or clinical outcomes in the original study groups (after the end of the study, the intervention was implemented in the whole population).

The theoretical model built considered two possible treatment options: "Hypertension Programme" or "Usual Care". We used a Markov model to allow for repeated cardiovascular events. Each cycle lasted 1 year. Costs and outcomes were tracked through-out patient's life-time. Even though this life-time perspective might be controversial, we chose to not exclude very old patients because of recent evidence of beneficial effects of hypertension treatment in this age group[12]. Nevertheless, we also explored the cost-effectiveness of the model considering different follow-up times.

**Figure 1:**
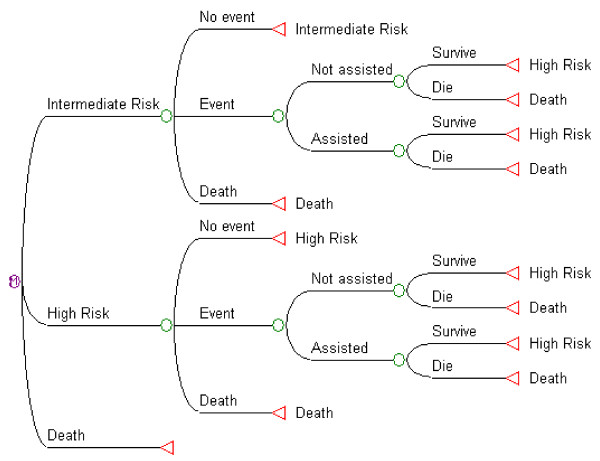
**Diagram of the Markov model for each treatment option (usual care; and hypertension programme)**. Basal cardiovascular risk status for patients could be intermediate risk (hypertension and age as only risk factors) or high risk (previous cardiovascular events and/or diabetes mellitus and/or other cardiovascular risk factors that gave a high risk prediction according to Framingham's algorithms). Both groups could follow the same alternatives. Patients started each 1-year cycle at the left hand, according to their basal cardiovascular risk. They could have an acute cardiovascular event or not or die from causes other than cardiovascular ones. Survival probabilities for an acute cardiovascular event depended on whether the patient received acute hospital care or not. Red triangles at the right hand show the starting point for the next one-year cycle: "Intermediate risk" continues in the intermediate risk group, "High risk" in the high risk group. Patients that died remained in that state until the end of the model run.

Independently of the treatment option chosen, patients could follow one of three different paths in each 1-year cycle, based on their transition probabilities (figure 1): a) Continue in the same health state without suffering any event; b) Have an acute cardiovascular event (acute myocardial infarction -AMI-, unstable angina -UA-, ischaemic stroke, haemorrhagic stroke, transient ischaemic attack -TIA-, heart failure -HF- and peripheral artery disease -PAD); or c) Die from causes other than cardiovascular disease. Patients who suffered a cardiovascular event could have acute hospital attention or not. All patients suffering an acute event could die during that year (cardiovascular death) or survive (at least for that year).

Transition probabilities depended on age and the general cardiovascular risk equation in the Framingham cohort study[25]. Because every patient had at least 65 years and hypertension at the start of the model, only two categories were included: intermediate and high risk (no patients with low risk). Irrespective of their basal risk, patients who survived after a cardiovascular event started a new cycle in the high risk group. Every patient that completed each cycle in any state other than death received 1 year of life gained (LYG). Yearly costs according to the treatment group were also computed. If a patient had suffered a cardiovascular event in that year, hospital expenditures were charged only for those who received hospital attention.

### Assumptions

Given that this model tried to capture a real-life scenario, we decided to include the probability of receiving hospital attention or not during an acute event. This is because of the relatively high proportion of patients with asymptomatic or atypical symptoms of cardiovascular events and/or sudden death. Patients assisted would have higher costs (related to hospital attention) and better survival outcome. These assumptions were the same for both groups (programme and usual care) because we considered that all hospitalized patients should have the same quality of health care in acute cardiovascular events.

### Sources of cost data

We conducted a micro-costing analysis of all resources involved in running this program (see table 1). Total cost per item was the unit cost times the quantity used. Capital costs were calculated as equivalent annual costs for a 5-year period using a 5% discount rate. We calculated all costs in 2006 Argentinean Pesos and adjusted them to 2010 values using the average consumer price index of different provinces from Argentina[26-31]. We report values in International Dollars using the purchasing power parity conversion rate suggested by the International Monetary Fund[32].

**Table 1:** Costs of the Hypertension Programme and Usual Care in 2010 International Dollars.

	Hypertension Programme			Usual care		
Concept	Unit cost #	Quantity *	Annual cost &	Unit cost #	Quantity *	Annual cost &
**Hypertension programme**						
**Labour**						
Physicians	15.73	3,168.00	49,820.80	NA	NA	NA
Fellows	10.27	4,752.00	48,808.07	NA	NA	NA
Monitors	5.29	15,744.00	83,272.20	NA	NA	NA
Education coordinator	24.22	204.00	4,940.97	NA	NA	NA
Educational workshops	20.76	564.03	11,708.14	NA	NA	NA
Secretary	6.60	528.00	3,485.35	NA	NA	NA
Nurse	7.93	13,305.60	105,507.51	NA	NA	NA
Epidemiologist	14.16	120.00	1,698.83	NA	NA	NA
**Labour subtotal**			**309,241.87**			**NA**
**Labour subtotal per patient**			**10.31**			**NA**
**Capital**						
Coordinator's furnishings	2,901.14	1.00	638.17	NA	NA	NA
Offices' furnishings	2,238.88	1.00	492.49	NA	NA	NA
Sphygmomanometer	90.50	7.00	139.36	NA	NA	NA
Coordinator's computers	1,703.98	5.00	1,874.16	NA	NA	NA
Offices' computers	1,703.98	4.20	1,574.29	NA	NA	NA
**Capital costs subtotal**			**4,718.48**			**NA**
**Capital cost subt per patient**			**0.16**			**NA**
**Land**						
Administrative office	112.09	7.50	10,088.39	NA	NA	NA
Medical office	112.09	7.50	10,088.39	NA	NA	NA
Support office	32.38	31.50	12,240.58	NA	NA	NA
Workshop space	16.61	564.03	9,366.51	NA	NA	NA
**Land subtotal**			**41,783.86**			**NA**
**Land suptotal per patient**			**1.39**			**NA**
**Resources**						
**Telephone**						
Effective call	0.12	7,959.96	971.57	NA	NA	NA
Ineffective call (non-response)	0.04	7,280.04	296.19	NA	NA	NA
**Telephone subtotal**			**1,267.76**			**NA**
**Telephone subtotal per patient**			**0.04**			**NA**
**Brochures**						
Brochures	1.07	10,000.00	10,711.13	NA	NA	NA
**Brochures subtotal**			**10,711.13**			**NA**
**Brochures subtotal per patient**			**0.36**			**NA**
**Surveillance software**						
Licence			4,151.60	NA	NA	NA
Hardware support			5,579.75	NA	NA	NA
Software maintenance	18.52	2,376.00	44,000.26	NA	NA	NA
Office	112.09	36.00	4,035.36	NA	NA	NA
Software development			11,588.72	NA	NA	NA
Server			3,397.26	NA	NA	NA
Computer			374.83	NA	NA	NA
**Software subtotal**			**73,127.78**			**NA**
**Software subtotal per patient**			**2.44**			**NA**
**Programme total**			**440,850.89**			**NA**
**Programme subtotal per patient**			**14.70**			**NA**
**Overhead costs**						
	1.98	8.14	16.07	1.98	7.64	15.08
**Overhead Subtotal (per patient)**			**16.07**			**15.08**
**Medical visits per patient §**						
Primary care physician	9.63	7.40	71.30	9.63	6.90	66.48
Specialist	9.63	0.74	7.13	9.63	0.74	7.13
Emergency ambulance service	1.45	12.00	17.44	1.45	12.00	17.44
**Medical visits subtotal**			**95.87**			**91.04**
**Consumption per patient**						
Drugs			198.99			160.48
Diagnostic/follow-up tests §			36.19			29.10
**Consumption subtotal per patient**			**235.18**			**189.58**
**Annual Total (per patient)**			**361.81**			**295.70**

# Labour: cost/hour; Capital, Brochures and Software: cost per item; Land: cost per m2 per month or per hour rented;

Telephone: cost per call; Overhead and Medical visit: cost per medical visit.

* Labour: number of hours/year; Capital, Brochures and Software: number of items; Land: total m2 or hours rented;

Telephone: number of calls; Overhead and Medical visit: number of medical visits.

& Capital costs are expressed in equivalent annual costs (5% discount rate, 5 years for all items except for software

development, 10 years).

§ No co-payments were charged.

NA: Not applicable

Regarding hospital costs for complications we could not use data from the same cohort studied in the original trial because of time frame restrictions and the subsequent implementation of the intervention in the whole population (including the usual care group). Thus, we decided to build a specific case-mix. We included all hospital admittances from cardiovascular events (AMI, UA, ischaemic stroke, haemorrhagic stroke, TIA, HF and PAD, coded using SNOMED-CT[33] in adult affiliates from 01/01/2006 to 12/31/2006. We tracked down costs (micro-costing) for each episode and then calculated the mean hospital cost per cardiovascular event as the average of all episodes during that period. Because the distribution was skewed to the right (as most cost data), we used the lognormal transformation for sensitivity analysis (see table 2).

**Table 2:** Variables for probabilistic sensitivity analysis: Costs in International Dollars for 2010.

Cost Variables	Base case	Distribution type	Distribution
**Usual care**			
Cost drugs/year	206.43	Lognormal	(4.36; 1.39)
Cost diagnostic/follow-up tests per year	29.10	Uniform	(20.37;37.83)
Number of medical visits	7.68	Lognormal	(1.68; 0.89)
**Hypertension programme**			
Cost drugs/year	216.55	Lognormal	(4.59; 1.26)
Cost diagnostic/follow-up tests per year	36.19	Uniform	(25.33; 47.04)
Cost programme	14.66	Uniform	(10.26; 19.06)
Number of medical visits	4.72	Lognormal	(1.16; 0.85)
**Common variables**			
Overhead cost (per visit)	1.98	Uniform	(1.39; 2.57)
Cost per medical visit	9.63	Uniform	(6.74; 12.52)
Cost ambulance/year	17.44	Uniform	(12.21;22.67)
Proportion of drugs coverage	0.70	Uniform	(0.40; 1.00)
Cost of cardiovascular event attention	10041.65	Lognormal	(8.24; 1.39)
Cost of diagnostic tests first year	117.78	Uniform	(82.44;153.11)

The discount rate used for the base-case was 5% for both costs and effectiveness, according to recommendations from the Panel on Cost-Effectiveness in Health and Medicine[34]. In the sensitivity analysis we considered up to a 12% discount rate according to suggestions from the World Bank for Latin America and Argentina[35].

### Sources of events and outcomes data

Annual rates of cardiovascular events for intermediate and high risk patients were calculated from the general cardiovascular risk equations in the Framingham cohort study[25]. Cardiovascular risk reduction from decreased SBP was calculated as suggested by a meta-analysis of individual data for one million adults in 61 prospective studies[36] using differences in final SBP levels between "Usual Care" and "Hypertension Programme" groups. As many references on outcomes did not report risks by gender, we decided to use average results and to not discriminate between sexes in the model.

Since we wanted the model to capture the cost-effectiveness as in a real-life setting, we considered those potential patients that would not receive health care attention during an acute cardiovascular event. Thus, we calculated the proportion of patients not assisted taking into account sudden deaths -from cardiovascular origin- and asymptomatic events -e.g. asymptomatic AMI- or atypical presentations[37,38]. Mortality data from cardiovascular events were taken from the same populations used to fit other probabilities in the model[39-42].

### Analysis

Since our aim was to inform decision makers from a third-party payer on the cost-effectiveness of these two approaches of hypertension treatment, we adopted this perspective to perform analyses. We did not have data from the original effectiveness study to also report results from a societal perspective. For the same reason, and budgetary constraints, we used life years gained (LYG) as an effectiveness measure and not quality-adjusted life years or other measure that considered health-state values. We did not extrapolate quality of life estimates from other populations due to clinically important differences in health states valuation in our region[43].

We calculated the incremental cost-effectiveness ratio between the different options using difference in costs in 2010 International Dollars divided by the difference in effectiveness in life years gained. All analyses were done with TreeAge Pro 2009 (TreeAge Software, Inc.).

We performed a one-way sensitivity analysis to explore the impact of each variable on results. A Tornado diagram analysis was used to assess the relative weight of each variable on overall uncertainty. We also explored variable uncertainty and the impact of simultaneous changes in variables included in the model with a probabilistic sensitivity analysis using Monte-Carlo simulations[44]. The model was run 100,000 times -iterations- taking different random samples of all variables used (except for discount rate). Tables 2 and 3 show variables used with its base case value and distribution.

**Table 3:** Variables for probabilistic sensitivity analysis: Outcomes

Probability variables	Base-case	Distribution type	Distribution	Reference
Reference population *				
Risk event in medium risk 65-74 years	0.0255	Uniform	(0.0223; 0.0285)	[25,48]
Risk event in medium risk 75+ years	0.0400	Uniform	(0.0300; 0.0500)	[25,48]
Risk event high risk group 65-74 years	0.0325	Uniform	(0.0300; 0.0350)	[25,48]
Risk event in high risk group 75+ years	0.2000	Uniform	(0.1500; 0.2500)	[25,48]
**Usual care group**				
Hazard ratio usual care group	0.6150	Normal	(0.6150; 0.0089)	[14,36]
Risk of event in middle risk group	a			
Risk of event in high risk group	b			
**Hypertension programme group**				
Hazard ratio programme group	0.5124	Normal	(0.5124; 0.0131)	[14,36]
Risk of event in middle risk group	c			
Risk of event in high risk group	d			
Scenarios of HR in programme group	0.5100	Uniform	(0.4500-0.5700)	[14,36]
**Common variables**				
Proportion initiate at medium risk	0.7000	Uniform	(0.0000;1.0000)	[14]
Starting age (years)	65	Uniform	(65-80)	
Risk of unrecognized event	0.3670	Uniform	(0.2500; 0.4000)	[38]
Risk of sudden death	0.1000	Uniform	(0.0600; 0.1400)	[37,50]
Mortality in assisted 65-74 years	0.1500	Uniform	(0.1000; 0.2000)	[39-42,46,47,50,52,53]
Mortality in assisted 75+ years	0.3000	Uniform	(0.2500; 0.3500)	[39-42,46,47,50,52,53]
Mortality in not assisted 65-74 years	0.3000	Uniform	(0.2000; 0.4000)	[39-42,46,47,50,52,53]
Mortality in not assisted 75+ years	0.6000	Uniform	(0.5500; 0.6500)	[39-42,46,47,50,52,53]

* A local reference population was used to calculate the risk reduction in both usual care and hypertension programme groups.

a) Risk of event in reference population (middle risk) × hazard ratio in usual care group

b) Risk of event in reference population (high risk) × hazard ratio in usual care group

c) Risk of event reference population (middle risk) × hazard ratio in programme group

d) Risk of event in reference population (high risk) × hazard ratio in programme group

Discount rate was considered a structural variable in the model. So, different analyses were performed with different discount rates, from 0 to 12%. A theoretical willingness to pay (WTP) threshold was set at Int$ 45,000, corresponding to 3 times the gross domestic product (GDP) of Argentina in International Dollars for 2010[32].

Due to its long-term perspective, model validation was performed according to Weinstein et al[45]. Face validity and verification were assessed during model construction, debugging and testing for internal consistency. Model results were consistent with observed data from mortality tables of populations were input data came from[46,47]. Corroboration was supported by the Markov model of the German hypertension treatment programme, although it had different health states and data sources[22]. Transparency and accreditation were sought through the publication of this research in an open access journal.

## Results

The base case showed that the least costly but least effective strategy was "Usual care". The "Hypertension Programme" had an incremental cost-effectiveness ratio (ICER) of 1,124 International Dollars per life-year gained (Int$/LYG). Results on total costs, effectiveness and incremental costs and effectiveness are shown in table 4.

**Table 4:** Results for the base case

Strategy	Mean Cost	Incremental cost	Mean Effect	Incremental effect	Average cost/effect	ICER
Usual care (IC95%)	$5,633.2 (2130 - 21027)		10.78 LYG (10.15 - 11.24)		522.44 $/LYG (163.92 - 2066.52)	
Programme (IC95%)	$5,828.5 (-9336 - 32499)	$195.3 (-11467 - 11472)	10.96 LYG (10.37 - 11.37)	0.18 LYG (0.08 - 0.29)	531.99 $/LYG (194.80 - 1936.86)	1,124.49 $/LYG (-75660 - 76230)

References: Mean effect: mean effectiveness; Incremental effect: incremental effectiveness; Average cost/eff: average cost-effectiveness; ICER: incremental cost-effectiveness; IC95%: 95% confidence interval; $: 2010 International Dollars; LYG: life-years gained. Discount rate 5%.

The variable that accounted for the majority of the uncertainty was the discount rate. It explained 91.7% of the uncertainty in the model. The next one was the starting age, explaining an extra 7.3%. Including the proportion of patients in the cohort starting with high cardiovascular risk, these 3 variables accounted for 99.7% of the overall uncertainty.

**Figure 2:**
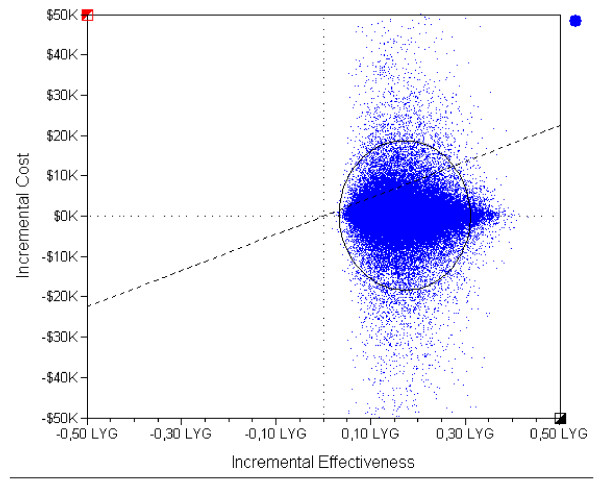
**Incremental cost-effectiveness scatter plot of "Hypertension Programme" versus "Usual care"**. Each blue dot represents the result of an iteration (a set of sampled variables) out of 100,000. The black circle represents the 95% confidence interval of results. The dashed diagonal shows the willingness-to-pay threshold of 45,000 Int$/LYG. Dotted lines mark 0 values for each axis. Incremental costs expressed per 1,000 (K) international dollars. Incremental effectiveness expressed in life years gained (LYG).

We performed a probabilistic sensitivity analysis including all variables in the model, except for discount rate (see tables 2 and 3). The ICER scatterplot of "Hypertension Programme" versus "Usual care" is shown in figure 2 for a discount rate of 5%. None of iterations showed less effectiveness. In 43% of them, "Hypertension Programme" was dominant. In addition, in 52% of cases the intervention had an ICER below a predefined WTP threshold of 45,000 Int$/LYG. Only 5% of iterations had an ICER above this threshold.

Being the discount rate the most sensitive variable, we ran the model and performed probabilistic sensitivity analyses for different values. Even at a discount rate of 12%, "Hypertension Programme" was dominant in 43% of cases. In 88.5% of times, the Programme was cost-effective.

**Figure 3:**
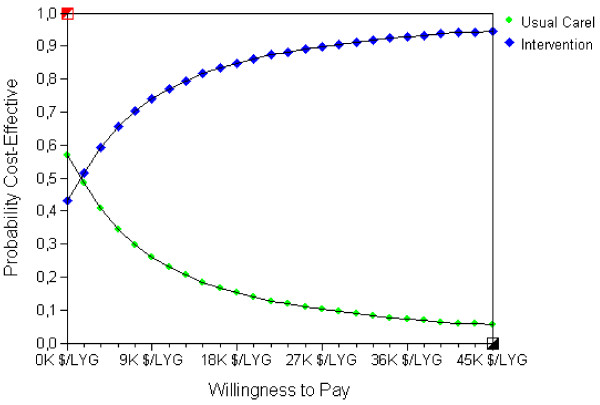
**Cost-effectiveness acceptability curve (CEAC) for treatment options**. Green circles depict "Usual care"; blue diamonds, "Hypertension Programme". Willingness to pay (WTP) is expressed per 1000 (K) international dollars per life-year gained ($/LYG). CEAC represent the probability for each intervention of being the most cost-effective option for different WTP thresholds. WTP is the maximum amount a society would be willing to pay, sacrifice or exchange for a good or service. The CEAC helps decision-makers to find the most probable cost-effective option according to the local WTP.

The cost-effectiveness acceptability curve (CEAC) shows the probability of the "Hypertension Programme" being cost-effective compared to "No Treatment" in a wide range of willingness to pay thresholds (figure 3). Considering a discount rate of 5%, at a WTP of 15,000 Int$/LYG (corresponding to Argentina's GDP for 2010), the "Hypertension Programme" had 82% probability of being cost-effective. At a WTP threshold of 45,000 Int$/LYG (3 times the GDP), the probability was 95%. See additional file 2: graphic S1 for CEAC for different discount rates.

## Discussion

This study showed that this "Hypertension Programme" was more effective than "Usual care" at a relatively small incremental cost. The base case result of ICER 1,124 Int$/LYG is highly cost-effective in our local context. Moreover, in 43% of 100,000 iterations performed in the probabilistic sensitivity analysis, "Hypertension Programme" was dominant (more effective and less costly). Overall, in 95% of cases, the programme was cost-effective.

This is the first study to include all the following aspects: original (short term) effectiveness data based on a primary source; hard outcome measurements; a long-term analysis; and a probabilistic sensitivity analysis. A literature review of four previous studies showed a combination of some methodological limitations in all of them: short-term analyses[21,24]; intermediate outcome measures[21,23]; a model based entirely on secondary sources[22]; or a biased sensitivity analysis[23].

In our model, the major determinant of uncertainty was the discount rate used. In general, benefits of hypertension treatments are seen several years after their start. As a result, the bigger the discount rate used, the lower the final benefit obtained. This is a common problem when considering cost-effectiveness of prevention programmes. Even though different discount rates produced different outputs, they would not significantly alter decision-making (see additional file 2: graphic S1). Nevertheless, a minimum 10 year-time horizon is needed.

The probabilistic sensitivity analysis evaluated uncertainty from all variables related to costs and outcomes used in the model. For example, even though we had exact costs for drug consumption (based on individual patients' drug purchase), we also included a variable for percentage of drug coverage by the payer. This variable tried to capture different economic burdens according to the percentage of coverage provided.

Regarding variables on transition probabilities for events and outcomes, we checked consistency of local and international data before fitting the model. We worked with different sources of systolic blood pressure levels[48,49] to try to detect possible differences in risks that could change outcomes in the model. Subtle differences among different data sources did not affect original cardiovascular risk probabilities.

Mortality data from cardiovascular events were taken from the same populations used to fit other probabilities in the model[39-42]. Given the lack of data regarding 1 year-mortality of untreated cardiovascular events, we decided to adjust these probabilities using national mortality tables (adjusted for age and cause of death) and observational studies [46,47,50] and to explore the range in the sensitivity analysis.

Our study's results are not directly comparable to previously published works[21,23,24] because they did not evaluate hard outcomes and/or have a long-term perspective. On the other hand, the German study[22] used a model that could allow broad comparisons. In general it can be said that they had findings similar to ours. This helps to corroborate results from both studies.

Of note, basal hypertension control in the usual care group from the study used to fit the model was high -60.4%- and mean basal blood pressure was 135/75 mm Hg[14]. In other settings, were basal control of hypertension is lower or the mean basal blood pressure is higher, a greater difference in effectiveness would be expected. For example, compared to a general elderly population in Argentina, the incremental effectiveness of "Hypertension Programme" would have been 1,22 LYG[48].

Even though the incremental effectiveness was relatively low for each patient, the model evaluated the effect of both types of hypertension treatment in all hypertensive patients in our population. Considering the impact of the programme in the 30,000 hypertensive patients in our setting, a total of 5,400 life years could be gained.

The model did not consider specific adverse events related to hypertension treatment for two reasons: 1) In previous studies, it was found that first-line anti-hypertensive drugs do not have more side effects than placebo[51]; and 2) to avoid double counting, because eventual costs and consequences of adverse events in hypertension treatment would be captured by the methodology used.

This study had some limitations. First, the effectiveness study used to compare treatment strategies was not a randomized controlled trial. It was impossible to perform one in our setting because of organizational restrictions (i.e. that could not prevent contamination of interventions between study groups). Nevertheless, "Hypertension Programme" and "Usual Care" groups had similar basal hypertension control in the originally published study, as mentioned above[14]. It did not have major methodological flaws, and its results were consistent with other studies[15-18]. Second, the study's perspective was not societal. Third, outcomes did not capture quality of life. Time-frame and budgetary restrictions prevented us from considering resource use from a societal perspective or from assessing utility measures during the original study. In addition, after the success of this demonstration study, all patients were treated according to the Hypertension Programme, precluding us from assessing any actual difference between groups. Given that the aim of the study was to inform decision makers from a third-party payer, the perspective adopted is all right. Nevertheless, a societal perspective might have given useful information and allowed analysis from other sectors. The lack of consideration of health state values (e.g. through QUALYs, etc.) is an important limitation. It is not possible to predict a possible influence of this fact. Effectiveness could have been lower (for example stroke survivors would have contributed with less than one QUALY per each year survived), but the bigger proportion of patients without cardiovascular events in the "Hypertension Programme" group could have summed more QUALYs overall. Thus, it would be interesting to address this important issue with a specific study designed "ad hoc" (to assess this effect). Fourth, only the effect of (different options of) hypertension treatment was evaluated. We chose this approach because our original experience only considered hypertension treatment. Nevertheless, a more integral approach, considering also treatment of other cardiovascular risk factors could have been adopted. This would have probably increased the effectiveness seen. Fifth, the study was based on urban populations from middle income and high income countries. Results should not be extrapolated to rural or low income populations. Finally, uncertainties inherent to the model were not explored. Because of the time-horizon chosen, it would have been impossible to avoid the use of a model, although different assumptions could have been made.

Notwithstanding, the study had several strengths. First, data on costs and effectiveness -intermediate outcomes- used to fit the model were local and at patient-level. Costs were evaluated in detail, and its real distribution was fitted in the model. Second, resources used were informed in appropriate physical units and valued in International Dollars to favour comparisons in other settings. Third, consistency of local and international data on events and mortality was checked before fitting the model. The slight differences observed did not modify model results. Fourth, a hard-outcome measure -mortality- was used. Fifth, the model was built to capture costs and outcomes of people with and without hospital attention during acute cardiovascular events as in a "real-life" scenario. Different assumptions can be made in different settings according to local access to health services and/or the rate of asymptomatic events. Finally, a probabilistic sensitivity analysis was performed with all variables included in the model. Results were robust under a wide range of assumptions.

## Conclusions

This is the first sound cost-effectiveness study to assess a comprehensive hypertension programme versus usual care. Its results showed that the "Hypertension Programme" was cost-effective against "Usual Care" for hypertension treatment and that its results were robust against wide assumptions.

Our study supports the idea that similar programmes could be the preferred strategy in countries and within health care systems where hypertension treatment for elderly patients is a standard practice.

## Abbreviations

AMI: acute myocardial infarction; HF: heart failure; HMO: health maintenance organization; ICER: incremental cost-effectiveness ratio; Int$: International Dollars; Int$/LYG: International Dollars per life-year gained; LYG: life-years gained; PAD: peripheral artery disease; PCP: primary care physician; PSA: probabilistic sensitivity analysis; SNOMED-CT: Systematized Nomenclature of Medicine-Clinical Terms; TIA: transient ischaemic attack; UA: unstable angina.

## Competing interests

The authors declare that they have no competing interests.

## Authors' contributions

All authors read and approved the final manuscript.

GP, CG, CP, SF, FGBQ, JC and ES contributed to study conception and design; GP, ER, GW and CA participated in the collection and assembly of data; GP, ER, GW, CA, CG, CP and ES contributed to analysis and interpretation of data; all authors participated in drafting of the article.

## Supplementary Material

Additional file 1 **Table S1 - Basal characteristics of patients in original effectiveness study**. Table showing basal clinical characteristics of the intervention and control groups in the original effectiveness study[14]. Click here for file

Additional file 2 **Graphic S1 - Cost-effectiveness acceptability curves for different discount rates**. Additional file 2, graphic S1: Cost-effectiveness acceptability curves for different discount rates: A) 0.0; B) 0.03; C) 0.07; D) 0.12. Each graph shows green circles for "Usual care" and blue diamonds for "Hypertension Programme". Willingness to pay expressed per 1000 (K) international dollars per life-year gained. Click here for file
