# Supplementary material for: Cost-effectiveness of a hypertension management programme in an elderly population: a Markov model
Source: Cost Eff Resour Alloc. 2011 Apr 5;9:4. doi: 10.1186/1478-7547-9-4 (PMC3084155; doi:10.1186/1478-7547-9-4)
Supplement: Additional file 1 — Table S1 - Basal characteristics of patients in original effectiveness study. Table showing basal clinical characteristics of the intervention and control groups in the original effectiveness study[14]. [file 1478-7547-9-4-S1.DOC]

### Additional file 1, table S1

**Table 5: Basal characteristics of patients in original effectiveness study.[14]**

| **Characteristics** | **Intervention group (Hypertension Programme)** | **Control group (Usual Care)** |
| --- | --- | --- |
| N | 250 | 250 |
| Women, n (%) | 162 (64.8) | 162 (64.8) |
| Mean age in years (range) | 73 (65-91) | 72 (66-88)^a^ |
| Mean blood pressure in mmHg, systolic (SD) / diastolic (SD) | 138 (20) / 75 (11) | 135 (19) / 75 (11) |
| Well-controlled patients, n (%) ^b^ | 141 (56.4) | 151 (60.4) |
| Patients with diabetes mellitus, n (%) | 36 (14.4) | 39 (15.6) |
| Mean body mass index, kg/m^2^ (SD) | 26.9 (4) | 27.7 (3.7) |
| Patients under antihypertensive pharmacological treatment, n (%) | 182 (73) | 175 (70) |
| Values are expressed as numbers (percentages or SD or range) as indicated.  ^a^ p<0.001 between groups  ^b^ Well controlled patients: systolic blood pressure <140 mmHg and diastolic blood pressure <90 mmHg.  p values not significant for other comparisons. | | |
