# Supplementary figures and images for: Cost-effectiveness of a hypertension management programme in an elderly population: a Markov model
Source: Cost Eff Resour Alloc. 2011 Apr 5;9:4. doi: 10.1186/1478-7547-9-4 (PMC3084155; doi:10.1186/1478-7547-9-4)

**A B**


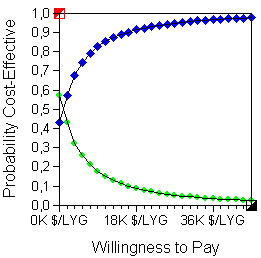

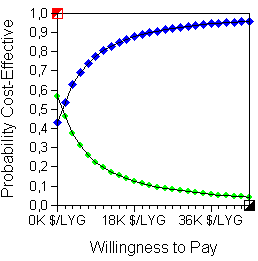


**C D**


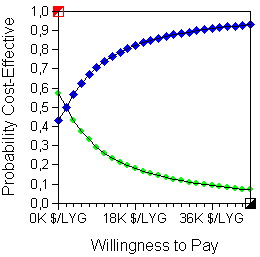

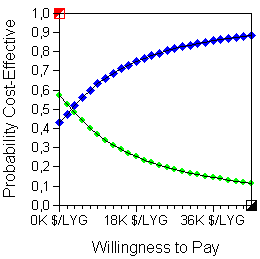

Supplement: Additional file 2 — Graphic S1 - Cost-effectiveness acceptability curves for different discount rates. Additional file 2, graphic S1: Cost-effectiveness acceptability curves for different discount rates: A) 0.0; B) 0.03; C) 0.07; D) 0.12. Each graph shows green circles for "Usual care" and blue diamonds for "Hypertension Programme". Willingness to pay expressed per 1000 (K) international dollars per life-year gained. [file 1478-7547-9-4-S2.DOC]
